# Deciphering the toxicity-effect relationship and action patterns of traditional Chinese medicines from a smart data perspective: a comprehensive review

**DOI:** 10.3389/fphar.2023.1278014

**Published:** 2023-10-16

**Authors:** Yubing Li, Xinyu Deng, Huiling Xiong, Qichao Hu, Yuan Chen, Wenwen Zhang, Xiao Ma, Yanling Zhao

**Affiliations:** ^1^ State Key Laboratory of Southwestern Chinese Medicine Resources, School of Pharmacy, Chengdu University of Traditional Chinese Medicine, Chengdu, China; ^2^ Department of Pharmacy, The Fifth Medical Center of the PLA General Hospital, Beijing, China

**Keywords:** toxicity, effect, relationship, traditional Chinese medicine, multiple omics

## Abstract

In Chinese medicine, the primary considerations revolve around toxicity and effect. The clinical goal is to achieve maximize effect while minimizing toxicity. Nevertheless, both clinical and experimental research has revealed a distinct relationship between these two patterns of action in toxic Traditional Chinese Medicines (TCM). These TCM often exhibit characteristic “double-sided” or “multi-faceted” features under varying pathological conditions, transitioning between effective and toxic roles. This complexity adds a layer of challenge to unraveling the ultimate objectives of Traditional Chinese medicine. To address this complexity, various hypotheses have been proposed to explain the toxicity and effect of Traditional Chinese Medicines. These hypotheses encompass the magic shrapnel theory for effect, the adverse outcome pathway framework, and the indirect toxic theory for toxicity. This review primarily focuses on high-, medium-, and low-toxicity Traditional Chinese Medicines as listed in Chinese Pharmacopoeia. It aims to elucidate the essential intrinsic mechanisms and elements contributing to their toxicity and effectiveness. The critical factors influencing the mechanisms of toxicity and effect are the optimal dosage and duration of TCM administration. However, unraveling the toxic-effect relationships in TCM presents a formidable challenge due to its multi-target and multi-pathway mechanisms of action. We propose the integration of multi-omics technology to comprehensively analyze the fundamental metabolites, mechanisms of action, and toxic effects of TCM. This comprehensive approach can provide valuable insights into the intricate relationship between the effect and toxicity of these TCM.

## Highlights

1. This review mainly focuses on representative high-, medium-, and low-toxicity traditional Chinese medicines (TCM) listed in Chinese Pharmacopoeia to demonstrate the crucial intrinsic mechanisms and elements that contribute to their toxicity and effectiveness.
2. The manuscript proposed the integration of multi-omics technology to analyze the fundamental metabolites, mechanisms of action, and toxic effects of TCM to comprehensively elucidate the relationship between their effect and toxicity.

## 1 Introduction

Toxicity and effect are two of the most important aspects of modern medicine. Toxicity, which reflects the potential risk of adverse effects, is a prerequisite for the use of medications. Effectiveness is an essential assurance that a medication can effectively treat a disease. Maintaining a balance between effect and toxicity is crucial in the clinical setting. The ultimate goal of medicine is to achieve a toxicity-effect relationship characterized by high effect and low toxicity. Interestingly, this concern regarding holistic health has been associated with the concept of “Harmony” in Traditional Chinese Medicine (TCM) for over 2,000 years. The disharmony between Yin and Yang is believed to be responsible for the onset and progression of several diseases. The curability of a disease is primarily attributed to the harmony between Chinese botanical drug and ailments.

Therefore, two decisive factors contribute to successful treatment progress: the medicine and disease/syndrome being treated. TCM employs a complex system for the preparation and administration of botanical drug. Any changes in parameters during this procedure could result in disparate outcomes. Aconitum, for example, is a commonly used toxic-effective medicine in TCM. Because of high toxicity potential, it must be processed properly based on traditional experiences or new technologies to ensure safety and effectiveness in treating cardiovascular diseases (Liu S. et al., 2017). Combining agents is another crucial factor in determining the effect and toxicity of medication; the classic example being the “eighteen incompatibles” theory. Research has demonstrated the effect of kansui in treating malignant pleural effusion. However, the effectiveness may be reduced, and potential toxicity may increase when licorice is used in combination with kansui at certain ratios (Shen et al., 2016a).

Treatment based on differentiation of disease is crucial for maintaining harmony. According to TCM, a toxic agent can be transformed into a beneficial medicine just with appropriate application. Arsenic trioxide (ATO) is a highly toxic mineral medication, even a small dose of which can result in severe central side effects or even death in healthy individuals. However, it has been found to be effective in treating many cancers, especially leukemia (Zhong et al., 2022). This example demonstrates the therapeutic applications of toxic medicines. In contrast, Polygonum multiflorum, a non-toxic TCM, has attracted attention for its potential toxicity and has been reported to cause liver damage when used to treat premature senility-related diseases. The specific population at high risk for *Polygonum multiflorum*-induced liver injury was previously unknown until a team from China identified the HLA-B*35:01 allele as a contributing factor (Li et al., 2019). These examples demonstrate that the different physical conditions or disease states is another decisive factor in achieving a toxicity-effect relationship characterized by high effect and low toxicity. Therefore, it is crucial to harmoniously identify the fundamental nature of this relationship.

## 2 Recognizing toxicity-effect relationship is challenging

**FIGURE 1:**
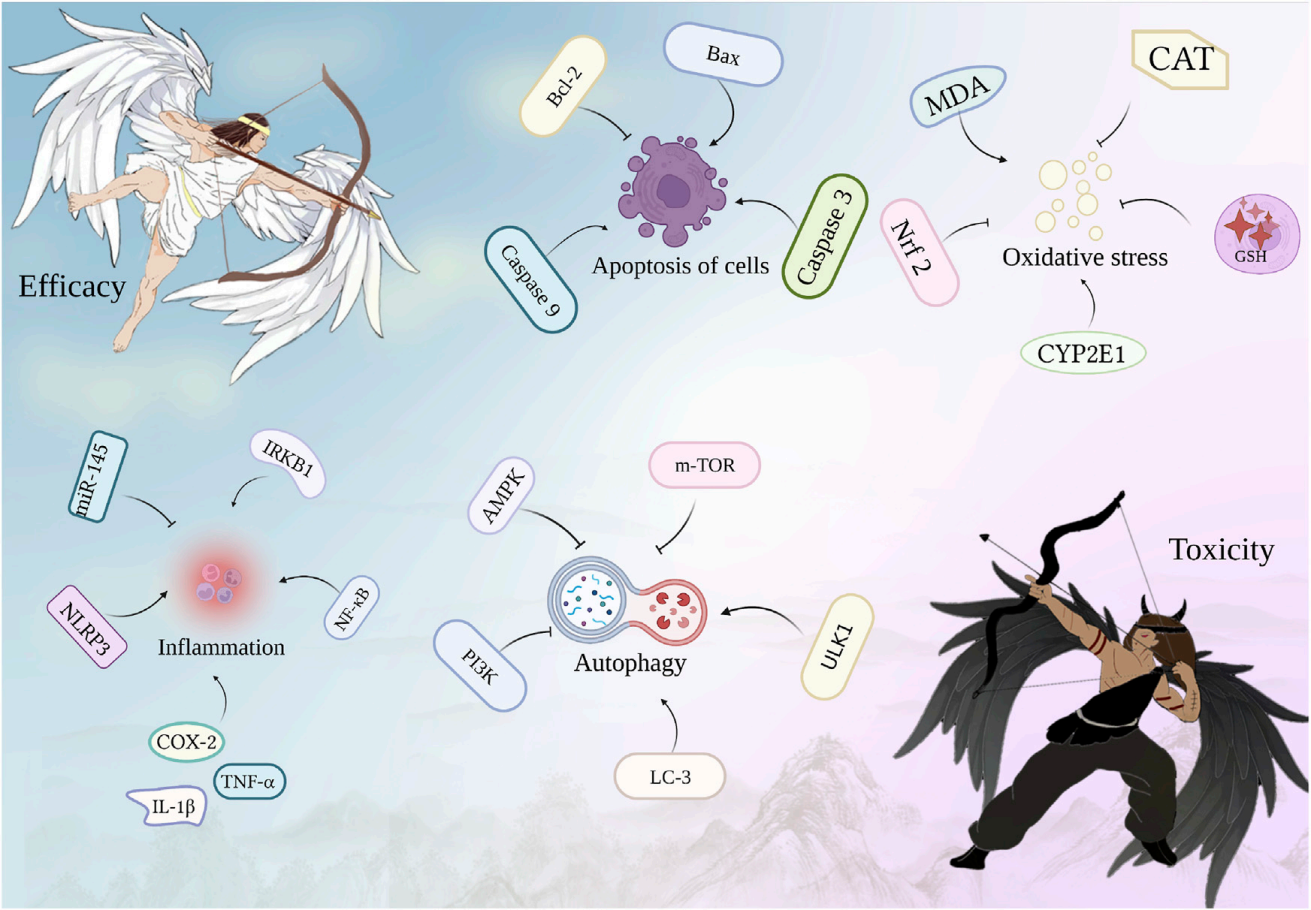
Pattern of action of the toxicity-effect relationship.

Recent studies have aimed to explore the scientific basis of the toxicity-effect relationship via metabolites-based and molecular approaches. However, identifying the core nature of this subject remains challenging for three reasons. First, botanical drug and their intrinsic metabolites are relatively complex. For example, at least 200 metabolites have been isolated from *Panax ginseng* C. A. Mey., a well-known botanical drug (Liu H. et al., 2020). Among these metabolites, at least six possible metabolites of ginsenoside Rb2 have been identified following administration (Karmazyn and Gan, 2021). Hence, the interactions (synergy or antagonism) between these metabolites can either enhance or inhibit their effects, leading to increased complexity. For instance, Li C. et al. (2022) identified six interactive metabolites in Xuebijing injection that exerted anti-sepsis action via pharmacokinetics and pharmacodynamics. The multi-metabolites and drug-combination pharmacokinetics further complicates this issue. Second, the paradigm of “one drug, one target” is shifting towards network analysis, which involves the use of multiple metabolites that target multiple signaling pathways or targets (Figure 1). Signal crosstalk increases the complexity of constitution. Tanshinone is a natural product derived from *Salvia miltiorrhiza* Bunge. Studies have shown that TLR‐4/NF‐κB, PI3K/Akt, and MAPK are core targets associated with various solid carcinomas and organ-related diseases (Li et al., 2020). Subtle variations in the dose can significantly alter the effect and toxicity of the signals. One study has revealed that *Rheum palmatum* L. doses ranging from 2 to 40 g/kg primarily exhibit hepatoprotective effects (Wang et al., 2011). This dose results in the alleviation of oxidative stress indices and other inflammatory markers. However, when the dose exceeds 80 mg/kg, it mainly demonstrates hepatotoxicity due to its involvement with targets associated with inflammatory reactions, oxidative stress, and cellular apoptosis. Third, the complexity of the effect and toxicity of botanical drug is increased by the whole body. Both western medicine and TCM agree that a set of factors are the primary causes of complex diseases (Li and Zhang, 2013; Mardinoglu et al., 2018). Western medicine is based on biological network models, whereas TCM relies on syndromic pathogens. These networks are considered integral systems comprising metabolic, transcriptional regulatory, protein-protein interaction, signaling, and co-expression networks. This provides a framework for a systematic understanding of how biological pathways are related to disease development. For example, autophagy has been shown to have opposing effects on liver injury and liver cancer. In liver injury, pretreatment with kaempferol significantly reduced survival and increased severe liver injury by promoting autophagy, whereas in hepatocellular carcinoma, kaempferol induced autophagy via the ER stress-CHOP pathway and regulated autophagy-related genes, Atg7 and siRNA, which induced cancer cell death, thus exerting an anti-cancer effect (Xiao et al., 2022). Therefore, kaempferol has distinct regulatory effects on autophagy at these two stages.

The complexity of substances or metabolites, multiple signals of agents, and the biological network of the body pose challenges in establishing a toxicity-effect relationship. This leads to distinct recognition of effect and toxicity.

## 3 Several representative theories regarding the toxicity and effect in TCM

### 3.1 Effect: from magic shrapnel to network analysis

Single botanical drug are rarely used in clinical practice. Instead, combinations of botanical drugs, referred to as formulas, are used to treat various diseases. Botanical drug formulas contain multiple active small-molecule metabolites that interact with different molecular pathways and targets, providing a theoretical basis for their use in treating various diseases. These active metabolites are highly concentrated, such as in shotgun shells, which have a broad range. Therefore, they have a high success rate in treating diseases, referred to as the “magic shrapnel” of Chinese botanical drug (Chen et al., 2006). This theory enhances the understanding of the materiality of botanical drug metabolites from the perspective of modern science, providing a novel approach for revealing their effectiveness mechanisms. However, the “magic shrapnel” theory only proposes a basic approach to treating diseases through botanical drug formulas but fails to provide an optimal solution for combining multiple active metabolites or botanical drugs.

Researchers have proposed the “metabolites theory” to optimize the combination effect of the active metabolites in TCM. This theory proposes the use of modern pharmaceutical preparation techniques to combine proven individual metabolites and create novel botanical drug formulas. This theory clarifies the various chemical metabolites of botanical drug and their fixed proportions, as well as facilitates the identification of direct targets and elucidation of the molecular mechanism of action. For example, when salvianolic acid A and B, the primary active substances from *S. miltiorrhiza* Bunge., were combined (in a 1:1 dose ratio), they inhibited the progression of renal interstitial fibrosis in rats by targeting the PDGF-C/PDGFR-α signaling pathway more effectively than either metabolite alone (Yao et al., 2022). Additionally, the combined use of phenolic acid from *S. miltiorrhiza* Bunge. and total saponin from *Panax notoginseng* (Burkill) F.H.Chen. in a 0.21:1 ratio inhibited neuronal apoptosis by regulating the phosphatidylinositol-3 kinase (PI3K)/protein kinase B (Akt) signaling pathway and mitigating cerebral ischemia/reperfusion injury in rats. This combination was more effective than either metabolite alone (Zheng et al., 2022). Furthermore, the combination of baicalein and baicalin from *Scutellaria baicalensis* Georgi. suppresses the proliferation of hepatocellular carcinoma cells by downregulating PD-L1 expression (Ke et al., 2019). These findings validate the reliability and usefulness of the “metabolites theory” and provide novel perspectives on the applications of TCM.

In the last decade, advancements in bioinformatics and their incorporation into TCM have led to a shift in the understanding of drug effect for treating complex diseases. The traditional concept of “one disease-one target-one drug” has been replaced by the concept of one active metabolite modulating “multiple targets-multiple pathways” to treat various diseases (Nogales et al., 2022). Network analysis technology uses nodes and connections to build a network model of interindividual connections, which converts the interactions of complex biological systems into networks. It further analyzes the composition and characteristics of these networks to achieve systematic identification of organisms, including the establishment of drug target libraries, disease gene libraries, “protein-protein interaction” networks, and “drug-target-disease” networks. This technique analyzes the multi-level and multi-faceted biological network relationships between “drug-target-disease” to predict the possible mechanism of action of drugs and provide important references for the discovery of pharmacological effect and mechanisms. Network analysis is widely used in botanical drug for target prediction and drug discovery in various diseases, including COVID-19 (Sun et al., 2023a; Jiang et al., 2023), cardiovascular diseases (Liu et al., 2022), diabetes (Sun et al., 2023b), and cancers (Chen and Han, 2022; Iksen et al., 2023). Although network analysis has redefined the application of Chinese medicine in the diagnosis and treatment of diseases, it has several limitations. Network analysis requires a comprehensive database; however, the reliability of the databases used for network analysis is a key concern.

### 3.2 Toxicity: adverse outcome pathway framework and indirect toxic theory

In 2010, researchers introduced the concept of an adverse outcome pathway (AOP) framework. AOP is a mechanism that connects molecular initiating events (MIEs) with deleterious outcomes via a series of sequential events, typically using key event relationships (KERs) (Kose et al., 2023). AOP integrates key events to link bioturbations at the molecular or cellular level with toxicity events to analyze damaging outcomes at the biological and population levels. It involves modularizing toxicity events at molecular, cellular, tissue, organ, individual, and population levels to provide coherent evidence of toxicity (Tollefsen et al., 2022). The application of AOP framework theory in TCM toxicity discovery is currently in its early stages. Multi-omics has advanced the field of toxicology and identified endogenous metabolites in TCM that are directly involved or regulate metabolic pathways, providing numerous MIEs for exploring TCM toxicity (Zhu et al., 2022).

In addition to the AOP framework, the indirect toxic theory has been proposed to elucidate the latent or potential adverse effects of TCM. It is similar to idiosyncratic toxicity but is more strongly influenced by external factors, such as preexisting diseases, syndrome status, and environmental elements. For example, *Epimedium brevicornu* Maxim. has traditionally been considered a non-toxic botanical drug for deficiency syndromes for many centuries. However, in recent years, authoritative studies have reported its effects on hepatic toxicity, particularly in patients with immune hyperfunctions compared to other patients. (Gao et al., 2020). Research has revealed that metabolites of *E. brevicornu* Maxim. can induce liver injury by specifically increasing NLRP3 inflammatory vesicles in response to certain triggers, thereby exacerbating the immune inflammatory response (Gao et al., 2020). Therefore, this specific toxicity is known as an indirect toxicity.

## 4 The toxicity-effect profile of toxic TCM

Recognizing the current toxicity-effect characteristics of botanical drug from the Chinese Pharmacopoeia (CP), an important source of clinically used medicines, is crucial. Toxic botanical drugs are classified into three categories based on their toxicity levels: strong, medium, and mild. This classification relies primarily on clinical observations and is characterized by toxic metabolites. According to commonly used and representative botanical drugs in the Chinese Pharmacopoeia, several botanical drugs have been investigated in detail (Table 1).

**TABLE 1:** Characteristics of specific toxic traditional Chinese medicines.

Traditional Chinese medicines	Toxicity and effect	Dosage	Mechanisms
*Mylabris phalerata* Pallas	Effect: hepatoprotective and tumor inhibiting	Oral 0.03–0.06 g in humans	Downregulate: JNK, ERK, PKC, and NF-κB
	Toxicity: apoptosis, leading to hepatocyte and renal toxicity	More than 0.06 g in humans	Upregulate: TLR4/NF-κB, ATF6/PERK, LC3, Beclin-1, Atg3, Atg4 and Atg7, TNF-α, IKK-α
Arsenic trioxide	Effect: treating acute promyelocytic leukemia	Oral 1–5 mg in humans	Upregulate: MAPK, PML-RARα, apoptosis
	Toxicity: leading to cardiotoxicity and hepatotoxicity	Oral 5–50 mg in humans	Upregulate: ROS, LDH、ROS、TNF-α、Bax
			Downregulate: AMPK/SIRT1/PGC-1α, p62-Keap 1-Nrf 2
*Aconitum carmichaelii* Debeaux	Effect: analgesic, cardiotonic and antiarrhythmic effects	Oral 3–15 g in humans	Upregulate: intracellular G protein/PI3K/PIP2 signaling pathway
			Downregulate: TRPV1 channels
	Toxicity: neurotoxic and cardiotoxic	Oral 10–16 g in humans	Upregulate: TNF-α-NLRP3, Bax、PARP2, NF-κB, Caspase3, Caspase 9
			Downregulate: BNiP3-dependent mitosis
*Euphorbia kansui* S. L. Liou ex S. B. Ho	Effect: treat various diseases, including tumors, leukemia, influenza, chronic bronchitis, and asthma	Oral 0.5–1.5 g in humans	Upregulate: TXA2/PGI2
			Downregulate: inflammatory response, NF-κB
	Toxicity: skin, oral, and gastrointestinal irritation, liver damage, and tumor-promoting toxicity	More than 1.5 g in humans	Upregulate: AQP2和AQP8, RAAS, apoptosis and inflammation
*Artemisia argyi* Levl. et Vant	Effect: anti-inflammatory, pathogen-resistant, analgesic, and anti-tumor activities	Oral 3–9 g in human	Upregulate: Bcl2
			Downregulate: TLR4/MyD88/NF-κB, ROS, Bax, NO, PGE2, TNF-α, IL-6
	Toxicity: convulsive on central nervous system, metabolic dysfunction in the liver cells, toxic jaundice, and hepatitis	Oral administration of about 100 g of AA can cause death	Downregulate: AhR signaling pathway, LPS/IL-1-mediated RXR, GABA receptors
*Evodia rutaecarpa* (Juss.) Benth	Effect: analgesic, anti-inflammatory, hepatoprotective, cardioprotective, and anticancer	Oral 2–5 g in humans	Upregulate: blocking cell cycle progression (G2/M phase) through activation of Cdc2/cell cycle protein B
			Downregulate: IFN-γ, NO, NF-κB, COX-2, PKB/Akt and p1S70k, HIF-6α
	Toxicity: cardiotoxicity and hepatotoxicity	Oral 30 g can be toxic in human	Upregulate: Erk1/2, Src, CDK8, and CK1e, MDA, CytC, apoptosis
			Downregulate: MnSOD
*Polygonum multiflorum* Thunb	Effect: anti-aging treatment	Oral 3–6 g in humans	Upregulate: NRF1/Keap1, HO-1, GSH, NQO2, SOD, CAT
			Downregulate: LPS/TLR4/NF-κB/MyD88
	Toxicity: hepatotoxicity	Oral 30 g can be toxic in human	Upregulate: MAPK, NF-κB, TNF-α, TLR4/MD2
*Panax ginseng* C. A. Mey	Effect: Strong heart and anti-shock; enhance learning and memory ability	5–10 g in humans	Upregulate: miR-873-5p
			Downregulate: HMOX12, neuronal cell apoptosis
	Toxicity: nervous excitement, resulting in insomnia, heightened agitation, and increased blood pressure, depression	Oral 4,000 mg/L Ginsenoside in rat cause liver injury	Upregulate: arachidonic acid, PGE2, leukotriene B4, apoptosis

### 4.1 The toxicity-effect profile of strong-toxic TCM

*Mylabris phalerata* Pallas., a well-known Traditional Chinese Medicine is listed as a highly toxic drug in the Chinese Pharmacopoeia (2020 Edition) with a recommended dosage of 0.03–0.06 g. However, cantharidin, the principal active metabolite extracted from *M. phalerata* Pallas., has demonstrated effect in treating various tumors by inducing apoptosis and cellular DNA chain rupture (Ren and Kinghorn, 2021). At standard doses, cantharidin exerts hepatoprotective effects by inhibiting liver tumorigenesis; however, it also induces some hepatotoxic effects. In terms of hepatoprotective effects, cantharidin inhibits cancer progression by inducing DNA damage via the JNK, ERK, PKC, and NF-κB pathways, and inhibiting their repair mechanisms in tumor cells (Naz et al., 2020). Studies have revealed that the toxic dose of cantharidin is close to the therapeutic dose. Reducing toxicity has become a primary concern in the application of cantharidin due to its narrow therapeutic window. Cantharidin can cause liver and kidney damage when administered under the therapeutic dose (1.0 mg/kg) (Cheng et al., 2022). Specifically, cantharidin induces toxicity in human hepatic L02 cells by activating endoplasmic reticulum (ER) stress, glucose-regulated protein 78 (GRP78), and apoptotic proteins. Transmission electron microscopy revealed ER expansion, autophagic vesicles, and apoptotic vesicles, which further induced apoptosis by regulating oxidative stress, ER stress, and autophagy, causing L02 cell hepatotoxicity. Continuous Cantharides treatment of LO2 cells activated the ATF6 and PERK pathways, which initiated downstream signalling pathways and induced apoptosis. Additionally, it upregulated the expression of proteins associated with activated autophagy, including LC3, Beclin-1, Atg3, Atg4, and Atg7. (Liu et al., 2020b). In addition, administration of 2.67 mg/kg cantharides to male SD rats resulted in increased expression levels of TNF-α protein and IKK-α gene. Whereas, inhibition of the TLR4/NF-κB pathway downregulate TNF-α expression, suggesting that TLR4/NF-κB is an important mechanism by which cantharides exerts hepatotoxicity (Wu et al., 2015). Astragalus polysaccharide (APS) can counteract the toxic effects of cantharidin by regulating primary bile acid biosynthesis and glycerol phospholipid metabolism. Additionally, APS can enhance cell survival by ameliorating cantharidin-induced oxidative stress (Huang et al., 2021). Research indicates that ginsenosides can effectively ameliorate cantharidin-induced acute kidney injury by mitigating the elevation of serum creatinine, urine protein, and urea nitrogen levels, as well as histological alterations in rats. The mechanism involves upregulating of BCL-2 to decrease cell apoptosis (Sacks et al., 2018). Additionally, clinical studies have shown that combining cantharidin with other botanical drugs and concoctions mitigates hepatotoxic effects. Shenmai injection combined with cantharidin significantly reduced the toxicity of cantharidin while maintaining its effect in patients with breast cancer (Wang et al., 2014). Furthermore, concoctions made from *M. phalerata Pallas, such as* alkali-treated or rice-fried combinations, are significantly less toxic than raw products. Among them, alkali-treated *M. phalerata Pallas* combined with sodium hydroxide were converted into sodium cantharidinate, which had reduced toxicity and improved antitumor activity. The heating process through the rice fried concoction reduced toxicity by sublimating free cantharidin in *M. phalerata Pallas*. (ZHANG et al., 2019).

In addition to *M. phalerata* Pallas., numerous highly toxic drugs have been used extensively in modern clinical practice. Arsenic trioxide (ATO), the principal metabolite of arsenic, has been used for thousands of years. Historically, it was used as a poison in ancient China, but more recently, its effectiveness in treating patients with relapsed or refractory acute promyelocytic leukemia has been demonstrated (Sanz et al., 2019). Despite its potential pharmacological benefits, its toxicity requires careful consideration. The prescribed daily oral intake within the safe limits is 1–5 mg, whereas the ingestion of rates ranging between 5 and 50 mg may result in human oral poisoning. According to scientific research, patients with acute promyelocytic leukemia exposed to a dose of 0.16 mg/kg for five consecutive days did not demonstrate significant cardiovascular toxicity (Yang et al., 2018). Regarding its therapeutic effect, ATO treatment induces phosphorylation of the PML protein via a MAP kinase pathway. This targets the PML element of PML-RARα, ultimately leading to apoptosis of acute promyelocytic leukemia cells. Nevertheless, research has demonstrated that ATO’s clinical usage is restricted due to its toxic properties towards both the liver and heart. Recent research suggests that the overabundance of ATO may cause cardiotoxic effects, such as oxidative stress, inflammation, and apoptosis. The specific mechanism involves inhibition of the AMPK/SIRT1/PGC-1α signaling pathway and effectively increaseing ROS generation and inflammatory signals (Han X. et al., 2022). Moreover, ATO can also accelerate the induction of oxidative stress, inflammatory response and cell apoptosis in cardiomyocytes by inhibiting the p62-Keap 1-Nrf 2 signaling pathway. These effects are mediated by increasing the levels of LDH, ROS, TNF-α, Bax, and Keap1 and reducing the levels of SOD, GSH, CAT, Bcl2, p62, and Nrf2 (Jia et al., 2021). In addition, in terms of hepatotoxicity, *Glycyrrhizae Radix et Rhizoma*, has been identified as a widely-used natural remedy in traditional Chinese medicine, that can regulate the effect and toxicity of other drugs. Specifically, its main metabolite, liquiritigenin, has been found to mitigate ATO-induced hepatotoxicity. Liquiritigenin improves ATO-induced cellular inflammatory responses and oxidative stress, regulates bile acid metabolism in the liver, and enhances liver cell autophagy by regulating the PI3K/AKT/mTOR pathway, thus protecting against ATO-induced liver damage (Zhang M. et al., 2021).

### 4.2 The toxicity-effect profile of medium-toxic TCM

*Aconitum carmichaelii* Debeaux., is another botanical drug of toxic in TCM. It primarily comprises various toxins including aconitine, mesaconitine, and neoaconitine. The consumption of 10–16 g of dried aconite can result in fatalities. The primary therapeutic benefits of this substance include analgesic effects, particularly in the treatment of cancer-related pain, paralysis of peripheral nerve endings when applied externally, and cardiotonic and antiarrhythmic effects on the cardiovascular system. Additionally, it exhibits both neurotoxic and cardiotoxic properties. Interestingly, the therapeutic and toxic doses of aconite are quite similar, with a range of 3–15 g and 10–16 g, respectively. A study revealed that aconitine, the primary toxic metabolite, is rapidly absorbed in the gastrointestinal tract and mainly affects the nervous and cardiovascular systems (Chan, 2009). Aconitine primarily affects neuronal ion channels and neurotransmitter energy metabolism, leading to nerve cell interference and neuronal apoptosis (Muroi et al., 1990). Excessive aconitine can cause myocardial electrophysiological disorders by continuously depolarizing the sodium ion channels in the myocardial membrane. This abnormal activation of sodium channels is the primary cause of aconitine-induced cardiotoxicity (Friese et al., 1997). Peng et al. (2020) found that adding aconitine at a concentration of 1.0 μmol/L can impede cardiomyocyte proliferation and trigger inflammation in H9c2 cells. Aconitine may induce cardiotoxicity by inhibiting BNiP3-dependent mitosis and affecting the TNF-α-NLRP3 signaling pathway (Peng et al., 2020). Aconitine exhibits anticancer properties by inhibiting the growth of Miapaca-1 and PANC-2016 cells and inducing apoptosis *in vitro* and *in vivo* in a time-and dose-dependent manner. The specific mechanism of action involves the upregulation of Bax, PARP2, cl-caspase-3, and cl-caspase-9 expression, as well as a reduction in Bcl-3 and NF-κB expression (Zhang et al., 2017). In addition, aconitine, a mixed-mutation regulatory ligand for capsaicinoids, may activate the intracellular G protein/PI3K/PIP2 signaling pathway by binding to endogenous cannabinoid receptors. This increases intracellular PIP2 levels, leading to the closure of TRPV1 channels and exerting analgesic effects (Xiao et al., 2019). Aconite and its metabolites have been found to exert cardioprotective effects, particularly in patients with heart failure. The potential improvement in cardiac mitochondrial function and maintenance of cardiac cellular homeostasis may be attributed to the presence of certain major alkaloids, such as aconitine, in aconite (Lu et al., 2017; Deng et al., 2019). In certain circumstances, physicians may administer high doses of aconite (120 g) for therapeutic purposes (Feng et al., 2014). The potential reduction in aconite toxicity during treatment aligns with the principles of TCM. However, the therapeutic range of aconite is limited because of the overlap of toxic and therapeutic doses. Clinical reports have focused on toxicity attenuation of aconite due to its poisonous nature. Chan (2014) found that extending the cooking time can transform alkaloids into non-toxic or less-toxic derivatives. Meanwhile, processed aconite shows lower toxicity than fresh aconite (Chan, 2014). In clinical applications, the combination of drugs can enhance the effect and mitigate aconite toxicity. Gallic acid can mitigate the toxicity of neoaconitine by inhibiting intracellular Ca^2+^ influx, restoring mitochondrial membrane potential, and reducing apoptosis (Han S. et al., 2022). In addition, berberine was found to prevent aconite-induced acute myocardial injury and arrhythmia, which may be related to the inhibition of aconitine-induced delayed depolarization and triggering activity (Chen et al., 2018).

*Euphorbia kansui* S. L. Liou ex S. B. Ho. (Kansui), a botanical drug exhibits an interesting balance between toxicity and effect in clinical application. This botanical drug is commonly used to treat various diseases, including tumors, leukemia, influenza, chronic bronchitis, and asthma (Shen et al., 2016b). However, Kansui shows toxicity at high doses, and long-term treatment can cause severe skin, oral, and gastrointestinal irritation, liver damage, and tumor-promoting toxicity, severely restricting its clinical application. Diterpenoids, the primary toxic metabolites of Kansui, cause inflammation, congestion, and increased peristalsis following oral administration. In CP, the therapeutic dose of Kansui ranges from 0.5 to 1.5 g. Kansui is used in TCM clinics for treating edema and ascites due to its potent purgative and detoxifying properties. It stimulates the contraction of gastrointestinal smooth muscle cells, thereby promoting intestinal peristalsis and reducing the bowel movement time (Zhang et al., 2020). Research has indicated that Kansui has the ability to enhance intestinal circulation by reinstating the relationship between TXA2/PGI2. This can help to decrease inflammation in patients who experience severe gastroenteritis. To achieve the desired anti-inflammatory effects, Kansui has demonstrated its potential to inhibit the activation of the transcription factor NF-κB in intestinal tissue. Vinegar is commonly used to reduce the side effects of Kansui owing to its toxicity. Zhang Q. et al. (2021) found that vinegar preparation can convert 3-O-(2′E,4′Z-decadienoyl)-20-O-acetylingenol (3-O-EZ), a primary active diterpenoid metabolite in Kansui, into ingenol. It reduces liver and gastrointestinal damage by improving abnormal pro-inflammatory cytokine and RAAS levels, and downregulating the expression of AQP2 and AQP8 (Zhang Q. et al., 2021). Furthermore, Bao et al. (2021) found that vinegar-processed Kansui could reduce toxicity by regulating anti- and pro-apoptotic mediators in the mitochondrial pathway and reducing inflammation. TCM theory suggests that the synergistic use of botanical drug can improve therapeutic outcomes while minimizing side effects or toxicity, a concept known as “TCM compatibility” (Shen et al., 2016b). The fruit of Ziziphus jujuba Mill. combined with Kansui can reduce the toxicity of Kansui. Metabolomics techniques were used to compare the toxicity of Kansui alone to that of Kansui co-administered with *Ziziphus jujuba* Mill. The results showed significant changes in metabolites indicating liver damage, renal lesions, and imbalance of intestinal microorganisms in the Kansui alone group; the Kansui and *Z. jujuba* Mill. combined group clearly showed a significant attenuation of toxicity (Liu et al., 2013).

### 4.3 The toxicity-effect profile of mild-toxic TCM

*Artemisia argyi* Levl. et Vant. (AA), a botanical drug with a history of over 2,000 years in TCM. AA demonstrates various pharmacological effects, including anti-inflammatory, pathogen-resistant, analgesic, and anti-tumor activities (Liu Y. et al., 2021). Although AA has therapeutic advantages, it also has toxic consequences, with its dosage being crucial in determining the association between toxicity and therapeutic effects. The CP records a common dosage of 3–9 g, and may be fatal when more than 100 g are taken. The volatile oil derived from AA has stimulatory and convulsant effects on the central nervous system. Additionally, AA may induce metabolic dysfunction in the liver cells, toxic jaundice, and hepatitis. *Artemisia argyi* Levl. et Vant. pretreatment has been found to significantly inhibit immune liver injury in mice by regulating serum transaminases, cytokine production, liver inflammation, and apoptosis. Further studies have shown that AA inhibits inflammation and apoptosis in immune liver injury by regulating the Bax/Bcl-2 and TLR4/MyD88/NF-κB signaling pathways (Yang et al., 2022). In addition, AA exerts anti-inflammatory effects by inhibiting the production of inflammatory mediators, such as NO, PGE2, TNF-α, and IL-6. This is achieved through negative regulation of JAK/STAT and ROS clearance, thereby reducing the protein and mRNA expression of iNOS and COX-2 (Chen et al., 2017). However, AA has been proven to be toxic, especially in relation to hepatotoxicity and neurotoxicity. Administration of a single dose of AA volatile oil (1.9 g/kg) for 6 h significantly increased the serum ALT and ALP levels. Additionally, the liver lobule structure was damaged by varying degrees of inflammation, suggesting the possibility of acute liver toxicity (Hongjie Liu et al., 2017). Subsequent research has revealed that AA-induced liver injury may implicate multiple pathways, including the crucial processes of inhibiting the AhR signaling pathway and the LPS/IL-1-mediated RXR functional pathway (Liu H. et al., 2017). The neurotoxicity is mainly due to thujone, one of the metabolites in AA. Thujone causes tonic-clonic seizures in a dose-dependent manner (Rivera et al., 2014). The mechanism involves dose-dependent inhibition of GABA receptors, leading to excitation and convulsions (Nemeth and Nguyen, 2020). However, when combined with borneol, AA can partially counteract the inhibitory effect of thujone on GABA production. This enhances the activity of GABA, thereby reducing fear and anxiety in mice and alleviating the neurotoxicity of AA (Liu Y. et al., 2021). Additionally, exceeding a dosage of 62.5 μg/mL AA resulted in embryonic developmental toxicity and cardiotoxicity in zebrafish, as evidenced by an increase in spontaneous convulsions and heart rate (Gu et al., 2022).

*Evodia rutaecarpa* (Juss.) Benth. (EF) was recorded as mildly toxic in CP due to the presence of several alkaloids, including evodiamine, rutecarpine, evocarpine, and dehydroevodiamine (Yang et al., 2006). The most appropriate therapeutic dose is 2–5 g, and may cause poisoning if taken for more than 30 g. Modern pharmacological studies have revealed that EF possesses analgesic, anti-inflammatory, hepatoprotective, cardioprotective, and anticancer properties. Inappropriate and excessive NO production is the main cause of the pathogenesis of various inflammatory diseases. EF inhibits NO production by interfering with the signaling events triggered by interferon- γ (IFN- γ), and inhibits the effect of NF-κB and the transcription of COX-2 to play an anti-inflammatory effect. In addition to this EF inhibits COX-6 expression and HIF-6α accumulation through PKB/Akt and p1S70k dephosphorylation, providing evidence for a novel mechanism of its anti-inflammatory activity (Liu et al., 2009). EF also exerts anticancer properties by blocking cell cycle progression (G2/M phase) through activation of Cdc2/cell cycle protein B, thereby inhibiting proliferation rate. EF exhibits apoptotic activity through activation of NF-κB, inhibits NF-κB-regulated gene products such as cyclin D1, X-chromosome interlocking IAP (XIAP), Bcl-2, and Bcl-XL, and increases expression of the apoptosis inducer Bax, which then induces apoptosis of the cancer cells by the cysteine asparaginase pathway (Takada et al., 2005). However, a single high dose of EF may result in cardiotoxicity and hepatotoxicity, exhibiting a “dose-time-toxicity” pattern. Researchers have found that both aqueous and alcoholic extracts of EF can induce liver dysfunction in rats through the involvement of Erk1/2, Src, CDK8, and CK1e. Moreover, EF aqueous extract alters rat liver metabolism, reduces MnSOD levels, increases MDA levels, and reduces ATP levels in the liver cells. This leads to ATP depletion and CytC release, ultimately triggering cell death signals (Cai et al., 2014). Zhang et al. (2022) identified evodiamine and rutaecarpine as primary cardiotoxic alkaloids in EF. EF aqueous extract (5.25 g/kg) disrupts the cGMP-PKG pathway and interferes with purine, lipid, and amino acid metabolism. This causes cardiac physiological dysfunction, abnormal electrocardiogram readings, and pathological injury (Zhang et al., 2022). Berberine has been found to regulate exogenous apoptosis of cardiomyocytes caused by EF through nuclear factor E2-related factor 2 (Nrf2) activation and non-reactive oxygen species-dependent pathways (Guan et al., 2020). Additionally, berberine inhibits the metastasis-promoting effect of evodiamine during the proliferation of gastric cancer cells. The mechanism involves inhibition of evodiamine-induced expression of IL-8 and the adhesion of evodiamine to HUVECs (Shi et al., 2013).

### 4.4 The toxicity-effect profile of “non-toxic” TCM

In recent years, *P. multiflorum* Thunb. (PM) and *P. ginseng* C. A. Mey. (PG) have gained attention owning to their potential toxicity. Although PM is commonly used as a tonic and an anti-aging treatment in clinical practice, its hepatotoxicity has been extensively documented. The best therapeutic dose recorded by the Chinese Pharmacopoeia is 3–6 g, and taking more than 30 g may cause poisoning effect. The toxicity of PM is associated with anthraquinones, which are similar to adrenocorticotropic hormones, and exhibit hepatotoxic effects. Moreover, the toxicity of PM depends on its dose, mode of administration, and preparation method. The severity of the toxic response is directly proportional to the PM dose. Li D. et al. (2022) identified 2,3,5,4′-tetrahydroxystilbene-2-O-β-D-glucopyranoside (TSG), emodin, emodin-8-o-β-d-Glucopyranoside (EMG), and physcion as the primary PM metabolites responsible for liver damage (Li D. et al., 2022). However, another study revealed only high-dose TSG combined with EMG induced liver lesions (Wang et al., 2022). On the one hand, it is crucial to implement appropriate processing techniques for mitigating the hepatotoxicity of this otherwise non-toxic medicine. The traditional processing method involves subjecting fresh PM to nine cycles of cooking and drying to achieve optimal effect and reduce toxicity (Liang et al., 2018). The “dose-time-toxicity” relationship of hepatotoxicity induced by a single dose of PM administered to mice showed that doses ranging from 5 to 20 g/kg caused significant damage to liver tissue. The hepatoprotective effects of PM can be attributed to its anti-inflammatory and antioxidant properties. The LPS/Toll-like receptor 4 (TLR4) pathway is considered an integral part of hepatic inflammation. In NAFLD rats, PM reduced inflammatory cytokine levels by inhibiting the LPS/TLR4/NF-κB/MyD88 pathway (Lin et al., 2015). And regulates the expression of HO-1, GSH, CAT, NQO2, SOD, SREBP-1c, and FAS by activating the NRF1/Keap1 pathway, thereby inhibiting oxidative stress and lipid accumulation in the NAFLD model (Hosseini et al., 2020). Conversely, the hepatotoxicity caused by emodin in PM at toxic doses is due to the activation of MAPK and NF-κB signaling pathways, which promote TNF-α production by binding to the TLR4/MD2 complex on the macrophage cell surface [Lin et al. (2015)]. PG, another non-toxic medicine, the best dose recommended by the Chinese Pharmacopoeia is 5–10 g, but the specific toxic dose is still poorly studied, shows indirect toxicity in clinical practice. Modern pharmacological studies have revealed that PG possesses strong heart and anti-shock; enhance learning and memory properties. Ginsenoside Rg 1 can inhibit neuronal cell apoptosis by regulating the expression of miR-873-5p in hippocampal neurons of rapidly aging dementia model-SAMP 8 mouse, and significantly reduce AD symptoms, providing a basis for further treatment of Alzheimer’s disease. Indirect toxicity is primarily caused by the pharmacological activity of bioactive substances, and is closely related to external factors. Studies from the previous century have identified toxic reactions resulting from the clinical misuse of PG. In a study conducted by Dr. Siegel, 133 participants who consumed ginseng for over a month exhibited symptoms of nervous excitement, resulting in insomnia, heightened agitation, and increased blood pressure. Some individuals experienced depression, loss of appetite, hypotension, and allergic reactions. According to Siegel, the effect of PG is due to the presence of damaranediol and triol glycosides in its metabolites (Siegel, 1979). Paik and Lee (2015) found that ginseng abuse and misuse can result in various adverse effects such as emotional disorders, allergies, cardiovascular and kidney damage, reproductive organ bleeding, gynecomastia, liver damage, hypertension, and reproductive disorders. Although ginsenosides are not significantly hepatotoxic at safe doses, overdose of ginsenosides (4,000 mg/L) inhibits the proliferation of normal hepatocytes and causes hepatocyte damage. High doses of rare ginsenosides increase the levels of the differential metabolite linoleic acid and thus affect the α-linolenic acid and linoleic acid metabolic pathways. Linoleic acid is synthesized into arachidonic acid, which is inflammatory at high concentrations, and is catalyzed by cyclooxygenase and lipoxygenase enzymes to produce the inflammatory factors prostaglandin PGE2 and leukotriene B4, which accelerate apoptosis by destabilizing cell membranes. In addition, ginsenosides cause embryotoxic and teratogenic effects in rodents (Mancuso and Santangelo, 2017). Therefore, non-toxic Traditional Chinese medicines should be evaluated for indirect toxicity to ensure their safety for clinical applications.

## 5 Smart data from multi-omics as the bridge for establishing the toxicity-effect relationship

### 5.1 Key elements of the toxicity-effect relationship

**FIGURE 2:**
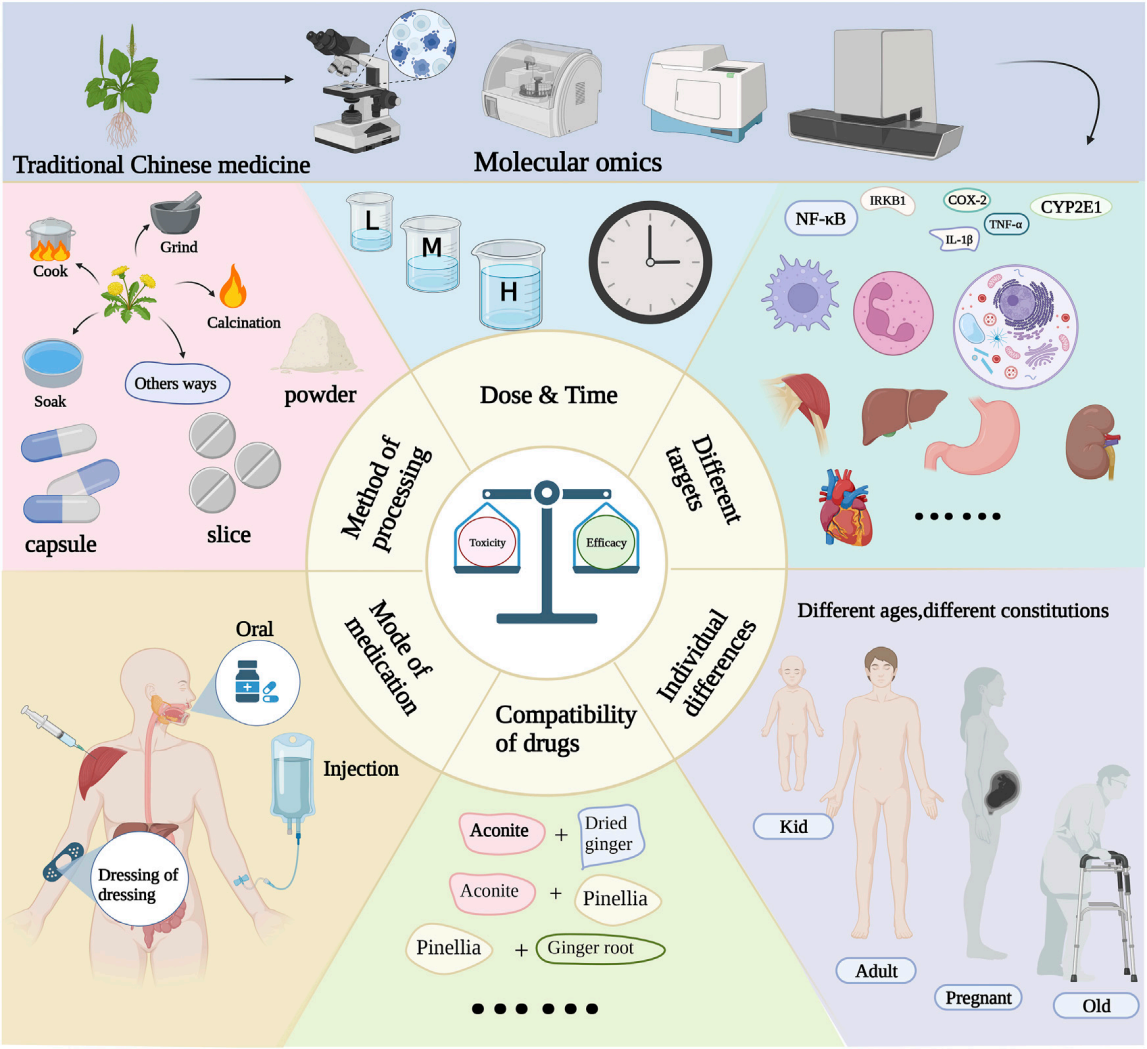
Multi-omics to explore the effect and toxicity of traditional Chinese medicines.

The toxicity-effect relationship relies on two fundamental factors: disease status and agent characteristics. The relationship between the mechanism of action of TCM and its biological response warrants further attention. Different organisms exhibit different reactions to the same drug. As an illustration, *A. carmichaelii* Debeaux. may induce severe toxic side effects, such as miscarriage, in pregnant women, while normal individuals can safely consume it at standard doses. Moreover, the characteristics of a drug can lead to toxic transformations, stemming from variations in its primary active metabolites before and after the botanical drug’s preparation. For instance, *A. carmichaelii* Debeaux. undergoes a process of concoction involving heating to volatilize aconitine, thereby reducing its toxic metabolites. Subsequently, it is combined with other botanical drug to enhance its effect and mitigate toxicity. It is worth noting that the method of administration can significantly influence the delicate balance between toxicity and effect. For instance, oral administration often results in low drug utilization, requiring substantial doses to reach the critical threshold for optimal effect. In contrast, intravenous administration exhibits significantly enhanced bioavailability, necessitating only a minimal dose to achieve the desired therapeutic effect. These variations in the route of administration also lead to distinct patterns of drug metabolism within the body, thereby altering the toxicodynamic relationship. Although complex biological reactions occur in the body, the multi-target and multi-pathway action of TCM adds complexity to the study of the toxicity-effect of TCM. Therefore, the scope and depth of this study should be enhanced to explore the mechanism of toxicity-effect. The current challenges in balancing toxicity-effect also provide new opportunities for future research, akin to a coin with two sides. Smart data analysis enables comprehensive evaluation of the multi-directional metabolic transformation of TCM in response to the body. The network simulation of drug-disease interactions can aid in predicting the mechanism of toxicity-effect, identifying novel biomarkers, establishing a comprehensive safety evaluation method, and achieving precision medicine. Thus, multi-omics technology may serve as the primary approach to elucidate the relationship between toxicity and effect of TCM in the future (Figure 2).

### 5.2 Smart data from multi-omics indicate precise evaluation

Multidisciplinary and cutting-edge technologies should be used to integrate and analyze the material basis, principle of action, and toxic-effect relationship of TCM and to reveal the relationship between the three aspects of drug effect: difference, body tolerance, and pharmacokinetic characteristics. This will enhance the scientific understanding of TCM, and its principle: “Medicine leads to no damage, also needs no excess in sick condition” (Bo Han et al., 2017). Using a holistic approach, multi-omics technologies use high-throughput analytical detection techniques in modern biological research systems to investigate the intrinsic connections between human tissue structure, function, biomolecules, and endogenous small molecules. The field encompasses metabolomics, proteomics, genomics, transcriptomics, and other related disciplines. Modern medicine uses omics technology to complete the histology of the human body and diseases, analyze disease occurrence, and identify drug treatment mechanisms (Hasin et al., 2017). This technology has provided comprehensive data in TCM to illustrate the disease status and medicinal characteristics. Furthermore, the integration of multi-omics facilitates the identification of therapeutic and toxic biomarkers for botanical drug post-administration. Integrating multiple omics can enhance disease diagnosis and treatment by identifying biomarkers, enhancing the comprehension of pathogenesis, and discovering therapeutic approaches, particularly in light of limited clinical data on mechanisms. We elucidated the advantages of omics approaches referring to articles that utilized omics techniques to explore the mechanisms of traditional Chinese medicine toxicity and effect.

#### 5.2.1 Multi-omics application of traditional Chinese medicine toxicity

Metabolomics as a commonly used method to reveal mechanisms of toxic effects can detect biomarkers at the metabolite level. The mechanism of cantharides induced hepatocyte toxicity was identified by LC-MS metabolomics method and 36 potential biomarkers were analyzed, among which 3 common biomarkers were used for cantharides induced hepatotoxicity in LO2 cells. These potential biomarkers were mainly related to nine metabolic pathways associated with cantharides hepatotoxicity, including tryptophan metabolism, purine metabolism, and glycine, serine and threonine metabolism. The results showed that cantharides exert its hepatotoxic effects through the regulation of single-carbon metabolism, tryptophan metabolism and purine metabolism (Liu et al., 2020c). In addition to this, endogenous bile acids could be quantified using UHPLC-MS/MS, and multivariate and metabolic pathway analysis of bile acids showed that scopolamine, bile acids and goose deoxycholic acid were the key biomarkers of cantharides induced hepatotoxicity (Cheng et al., 2022).

Transcriptomics is also commonly used to reveal the mechanism of action of toxic botanical drug. For example, rhodopsin exhibits significant hepatotoxic and nephrotoxic effects. Wu et al. (2018) analyzed 55 human liver and kidney samples using integrated metabolomic and transcriptomic data. These findings suggest that UDP-glucuronosyltransferase 2B7 (UGT2B7) plays a significant role in rhodopsin metabolism by serving as the primary enzyme responsible for glucuronidation. Subsequent research has indicated that the long-term use of rhodopsin decreases UGT2B7 activity, causing rhodopsin accumulation and consequent hepatotoxicity. Upregulation of multidrug resistance protein-2 (MRP2) in the liver following rhodopsin administration could mitigate rhodopsin-induced hepatotoxicity. UGT2B7 and MRP2 are hypothesized to be key proteins that influence the toxicity-effect relationship of rhodopsin (Wu et al., 2018). Zhao et al. (2018) studied the hepatotoxicity of Huang Yao Zi in rats using proteomic and metabolomic approaches. Integrative histological data showed dose-dependent alterations in 1,366 proteins and 58 metabolites, revealing hepatotoxic metabolic pathways and biomarkers (Zhao et al., 2018).

The mechanism of action of *P. multiflorum* Thunb as a toxic Chinese medicine was investigated by proteomics techniques to investigate the *P. multiflorum* Thunb-induced hepatotoxicity in rats. A total of 588 differential proteins were identified in the administered group, of which 300 were downregulated and 288 were upregulated (Lin et al., 2017). Then screened for potential hepatotoxicity biomarkers, NADH dehydrogenase 2 subunit C2 (Ndufc3) and NADH dehydrogenase flavoprotein 3 (Ndufv2) were co-expressed in the administered group. Further pathway analysis showed that these differential proteins are involved in spliceosome digestion and uptake, metabolism, oxidative phosphorylation and carbohydrate pathways, apoptosis and thyroid hormone signaling pathways. It was revealed that hepatotoxicity caused by *P. multiflorum* Thunb was mainly associated with abnormalities in the mitochondria-related oxidative phosphorylation pathway, leading to apoptosis.

#### 5.2.2 Multi-omics application of traditional Chinese medicine effect

As a typical “hot” Chinese medicine, aconite has been widely used for the treatment of cold-related diseases for thousands of years, but the critical mechanism of its promotion of thermogenesis has not been completely solved. Liu J. et al. (2021) used multi-omics techniques to investigate the specific mechanisms of the intestinal microbiota and bile acid metabolism in enhancing thermogenesis. The results showed that the abundance of Muribaculaceae, Ruminococcaceae, Desulfovibrionaceae, Enterococcaceae, and Lachnospiraceae bacterial population in the model group was significantly decreased. Further GO functional enrichment analysis showed that aconite restored the functional decline in energy metabolism, carbohydrate digestion and absorption, and PPAR signaling pathway in the model group. It was shown that all the above-mentioned species increased significantly after aconite administration and that aconite treatment improved the disturbance of intestinal flora as well as restored the function of intestinal microbiota involved in thermogenesis. Moreover, by targeting bile acid metabolism, it was found that aconite administration significantly restored the downregulation of the total BAs, total PBAs, and total SBAs in the model group, suggesting that the thermogenesis-promoting effect of aconite was closely related to the increase of bile acid.

Meanwhile, transcriptomics can also be used to explore the mechanism of medication action. The transcriptomics was used to investigate the mechanism of aconite in the treatment of cardiac hypertrophy. 1,284 differentially expressed genes were upregulated and 54 were downregulated between the aconite administration group and the control group (Xiaofang Xie et al., 2022). The differential gene pathway enrichment analysis showed that aconite mainly alleviated the symptoms of cardiac hypertrophy by activating ABC transporters and PI3K-Akt signaling pathway and inhibiting the expression of mTOR, JAK, STATA, MAPK, TNF-α, Calcium, Wnt, Ras and p53 signaling pathway genes.

Although Arsenic trioxide is a highly toxic botanical drug, it is commonly applied in clinical practice for the treatment of leukemia. Wang et al. (2017) used HL-60 cell line to examine the effect of different concentrations of Arsenic trioxide on cell-induced apoptosis and used proteomics to reveal the protein targets that regulate apoptosis. A total of 102 differential proteins were detected in the protein spectrum, of which 75 were upregulated and 27 downregulated in HL- 60 cells. These proteins are mainly metabolic enzymes or associated with cell cycle regulation, cell proliferation and apoptosis, signal transduction and DNA repair. Among these proteins, the expression of CH60, AT1A1, ANXA6, and EF2 was increased, and the expression of ATPA, MYO1G, and RS3 was decreased. These proteins play a role in promoting Arsenic trioxide-induced apoptosis in HL-3 cells, providing a new potential target for the clinical application of Arsenic trioxide in the treatment of leukemia.

### 5.3 Limitation

Utilizing omics data for investigating the correlation between the toxicity and effect of traditional Chinese medicine represents a highly efficacious approach. However, it is imperative to acknowledge the associated limitations and exercise prudence in their utilization and interpretation. The following are some constraints inherent in omics data: 1. The reliability of omics data hinges upon the source’s origin and data quality. Errors or noise present in the foundational data can exert a profound influence on subsequent analysis and interpretation; 2. The significance of sample size and diversity cannot be overstated in omics studies. A restricted sample size or samples that fail to adequately represent the population’s diversity can yield results with limited generalizability; 3. While computational studies can serve as an auspicious point of departure for metabolite identification, they remain inherently predictive in nature. Consequently, research pertaining to metabolites, particularly if the identified metabolites are recognized as pan-assay interference substances, may engender spurious positive outcomes.

## 6 Conclusion

Safety and effect are crucial considerations in TCM research. Ensuring the safe use of TCM is a major requirement in the fields of medicine and health. The safety of TCM has been questioned despite the increasing global recognition of its effectiveness. Between 2018 and 2020, TCM accounted for an average of 13.6% of reported adverse reactions/events compared with other drugs. The number of reported adverse reactions/events for TCM during this period was 1.597, 1.635, and 1.798 million, respectively. These data highlight the necessity of discussing the need for more precise applications of botanical drugs. Multi-omics technologies have established a correlation between body health status and medicine by providing smart data for assessment. The integration strategy plays a significant role in establishing the toxicity-effect relationship. Thus, the toxicity-effect relationship can summarize the effect, toxicity, and dynamic variations of TCM, encompassing factors such as dose, variety, processing, and compatibility.
